# Eukaryotes in the gut microbiota in myalgic encephalomyelitis/chronic fatigue syndrome

**DOI:** 10.7717/peerj.4282

**Published:** 2018-01-22

**Authors:** Alexandra H. Mandarano, Ludovic Giloteaux, Betsy A. Keller, Susan M. Levine, Maureen R. Hanson

**Affiliations:** 1 Department of Molecular Biology & Genetics, Cornell University, Ithaca, NY, United States of America; 2 Department of Exercise & Sport Sciences, Ithaca College, Ithaca, NY, United States of America

**Keywords:** Microbiome, Fungi, 18S, Eukaryotic, Inflammation, Sequencing, Diversity, ME/CFS, Fecal, Gastrointestinal

## Abstract

Patients with myalgic encephalomyelitis/chronic fatigue syndrome (ME/CFS) often suffer from gastrointestinal symptoms and many are diagnosed with irritable bowel syndrome (IBS). Previous studies, including from our laboratory, have demonstrated that the ME/CFS gut bacterial composition is altered and less diverse when compared to healthy individuals. Patients have increased biomarkers of inflammation and leaky gut syndrome. To further investigate dysbiosis in the ME/CFS gut microbiome, we sought to characterize the eukaryotes present in the gut of 49 individuals with ME/CFS and 39 healthy controls. Using 18S rRNA sequencing, we have identified eukaryotes in stool samples of 17 healthy individuals and 17 ME/CFS patients. Our analysis demonstrates a small, nonsignificant decrease in eukaryotic diversity in ME/CFS patients compared to healthy individuals. In addition, ME/CFS patients show a nonsignificant increase in the ratio of fungal phyla *Basidiomycota* to *Ascomycota*, which is consistent with ongoing inflammation in ME/CFS. We did not identify specific eukaryotic taxa that are associated with ME/CFS disease status.

## Introduction

Myalgic encephalomyelitis/chronic fatigue syndrome (ME/CFS) is a disease without identified causes or mechanisms, characterized by symptoms of profound fatigue, pain, cognitive impairment, post-exertional malaise and inflammation (Institute of Medicine, 2015). Patients are commonly diagnosed with ME/CFS following a flu-like illness, implicating an inciting pathogen in the disease. In some cases, patients became ill following a diagnosed viral, bacterial or protozoan infection. Patients often complain of gastrointestinal symptoms, such as stomach pain, diarrhea, and nausea. Many patients are diagnosed with gastrointestinal disorders such as irritable bowel syndrome (IBS) (Institute of Medicine, 2015; Maes et al., 2014).

In a recent investigation of the ME/CFS gut microbiome, our laboratory used 16S ribosomal RNA sequencing to demonstrate a decrease in the overall diversity of gut prokaryotes in patients with ME/CFS compared to healthy controls (Giloteaux et al., 2016). Patients had decreased abundance of several anti-inflammatory species and increased abundance of several pro-inflammatory species of bacteria (Giloteaux et al., 2016). Specifically, species belonging to the *Firmicutes* phylum were reduced and *Proteobacteria* abundance was increased in ME/CFS patients (Giloteaux et al., 2016). Assays for blood markers of inflammation revealed significant increased lipopolysaccharide (LPS), LPS binding protein (LBP) and soluble CD14 (sCD14) (Giloteaux et al., 2016). Previous studies of the ME/CFS gut microbiota using both culture-based and high throughput sequencing methods have revealed differences in bacterial composition between patients and healthy controls (Armstrong et al., 2017; Frémont et al., 2013; Navaneetharaja et al., 2016; Sheedy et al., 2009; Shukla et al., 2015). Evidence of increased gut permeability correlating with inflammatory markers and gastrointestinal discomfort was previously demonstrated in ME/CFS patients via IgA and IgM responses to LPS (Maes et al., 2014; Maes, Mihaylova & Leunis, 2007; Maes et al., 2012). Recent work by Nagy-Szakal et al. (2017) identified further differences in gut bacterial composition in patients with ME/CFS with and without IBS compared to healthy controls. Several studies have documented bacteriotherapy or oral probiotic treatment as alleviating ME/CFS symptoms or have linked symptoms to bacterial composition (Borody, Nowak & Finlayson, 2012; Rao et al., 2009; Sullivan et al., 2005; Wallis et al., 2016).

As the significant role of the gut microbiome in human health has become clearer, most research has focused on prokaryotic residents in the gut. However, the gut microbiota also consists of populations of eukaryotes and viruses (Clemente et al., 2012). Eukaryotes in the gut include protozoa and fungi, which can be commensal or pathogenic organisms (Hamad, Raoult & Bittar, 2016). For example, *Blastocystis*, historically linked to several human diseases, is now known to be present both commonly and commensally in humans (Scanlan & Marchesi, 2008). Eukaryotes interact with prokaryotes present in the gut and prokaryotic composition can influence the pathogenicity of eukaryotes (Chudnovskiy et al., 2016; Clemente et al., 2012). In some cases, gut eukaryotes are pathogenic only when the immune system is compromised (Clemente et al., 2012; Mason et al., 2012; Underhill & Iliev, 2014). Eukaryotes in the gut have limited similarity across healthy individuals (Li et al., 2014).

Alterations in gut eukaryotes have been linked to human autoimmune diseases, obesity, allergies, inflammatory bowel diseases (IBD), and diabetes (Clemente et al., 2012). In IBD, there is evidence of elevated antibodies against *Saccharomyces cerevisiae* and eukaryotic dysbiosis, which has been linked to inflammation (Chehoud et al., 2015; Li et al., 2014; Mukherjee et al., 2015; Ott et al., 2008; Seibold et al., 2001; Sokol et al., 2017). Pathogenicity of the protozoan *Entamoeba hystolytica* is influenced by inflammation and protozoan infections can also induce release of IgE and inflammatory cytokines (Chudnovskiy et al., 2016; Clemente et al., 2012; Moonah, Jiang & Jr, 2013).

ME/CFS has been previously linked to a possible eukaryotic pathogen. In particular, giardiasis was implicated in ME/CFS outbreaks in New Zealand in 1984 and California in 1985 (Levine et al., 1992; Snow, 2002). Following an outbreak of giardiasis in Norway in 2004, at least 5% of infected individuals developed ME/CFS (Naess et al., 2012). A link between ME/CFS and a protozoan infection such as *Blastocystis* or *Dientamoeba* has been suggested (Dunwell, 2013). An investigation of *Candida albicans* in stool samples found increased abundance in ME/CFS patients during the acute phase of the illness versus remission (Evengard et al., 2007). A recent study using culture-based methods investigated total yeast abundance in ME/CFS stool samples and found a nonsignificant decrease compared to healthy individuals (Armstrong et al., 2017).

Studies of eukaryotes in the gut have been limited in part by available methods and databases. Eukaryotes can be identified by the variable regions of their 18S rRNA gene, just as prokaryotes can be identified by 16S sequencing. Based on previous findings of dysbiosis and inflammation in ME/CFS, we hypothesized that the gut eukaryotic community may be altered in ME/CFS and could provide additional clues and potential diagnostic markers for this disease. To characterize gut eukaryote composition and investigate a possible link between gut eukaryotes and ME/CFS, we sought to identify eukaryotes present in the gut of both ME/CFS patients and healthy individuals. To accomplish this, we amplified and sequenced the V9 region of the 18S rRNA gene in both patients and healthy controls and analyzed the results for taxa and diversity differences.

## Materials and Methods

### Subjects and sample collection

ME/CFS patients and healthy controls were recruited by Dr. Susan Levine, M.D., in New York City. Patients were diagnosed with ME/CFS under the Fukuda Criteria (Fukuda et al., 1994). Additional healthy controls were recruited at Ithaca College by Dr. Betsy Keller. Individuals with a known acute illness or chronic infectious disease were excluded from the study. Written consent was obtained from all participants. Stool samples were collected by subjects at home and refrigerated in RNAlater. Following overnight shipment to Cornell University, the samples were stored in aliquots at −80 °C. For all subjects, BMI, age, sex, and gastrointestinal symptom information was collected. Patients also completed Bell’s disability scale (Bell, 1994). All protocols involving human subjects were approved by the Cornell University Institutional Review Board (#1012001855).

### DNA extraction and 18S amplification

Genomic DNA was isolated from stool samples of 39 healthy controls and 49 ME/CFS patients according to a modified protocol from Iliev et al. (2012). Briefly, stool pellets were resuspended in lyticase and lyticase buffer and incubated at 30 °C for 30 min, then spun at 4,000 rpm for 5 min. After removing the supernatant, pellets were re-suspended in stool DNA stabilizer (B Bridge International), homogenized on a bead beater with 0.1 mm and 0.5 mm beads, heated at 95 °C for 10 min, vortexed for 5 s, cooled on ice for 5 min, and spun at 15,000 rpm for 1 min. The remaining extraction steps were conducted with the QIAmp DNA Mini Kit (Qiagen, Valencia, CA, USA). The V9 region of the 18S rRNA gene was amplified using Illumina_Euk_1391f universal forward primer and individually barcoded Illumina EukBr reverse primers, according to the Earth Microbiome protocol (Earth Microbiome Project, 2017). Products were checked via gel electrophoresis for an approximate size of 200 base pairs and purified using Mag-Bind EZ-pure beads (Omega Bio-Tek, Norcross, GA, USA). DNA concentration was determined using QuantIT PicoGreen dsDNA kit (Invitrogen, Carlsbad, CA, USA) and 100 ng of each product was pooled for sequencing. Subjects with concentration too low to pool 100 ng were eliminated, leaving 29 controls and 35 ME/CFS patients. Five water controls from PCR amplification were included in sequencing to serve as negative controls. Pooled products were sequenced in a 2 × 250 bp paired-end run on the Illumina MiSeq platform at the Cornell Biotechnology Resource Center Genomics Facility. The sequence data will be available in the European Nucleotide Archive of the European Bioinformatics Institute under accession number PRJEB23827.

### Sequence analysis

Sequencing data was analyzed with Quantitative Insights into Microbial Ecology (QIIME) software (1.9.1). Due to poor quality in the second read, analysis was carried out solely using the data from read one. Sequences were split by barcode, the quality threshold was set at 25 and the reverse primer and downstream sequence were removed. Operational Taxonomic Units (OTUs) were picked using the open-reference method at a similarity of 97%. Taxonomy was assigned using BLAST against the SILVA 119 database with a minimum OTU size of 5 and an e-value of e−50. Taxa corresponding to archaea, bacteria, plant or mammal were filtered from the dataset, as were any OTUs identified in negative controls. 19 healthy controls and 23 ME/CFS patient samples contained OTUs and were retained for further analysis.

### Taxa abundance analysis

To compare specific abundances between patients and controls, taxa were considered present in an individual sample only when represented by at least 5 OTUs. Following removal of OTUs represented by fewer than five sequences in a given sample, data remained from 17 ME/CFS patients and 17 healthy controls. Statistical comparisons of taxa abundance were conducted in both QIIME and in R. Normality of data was checked using a Kolmogorov–Smirnov test. A Wilcoxon rank-sum test was used to test differences in taxa abundance between ME/CFS patients and healthy controls.

### Diversity comparisons

Sequences were aligned in QIIME using PyNAST and a minimum identity of 60%. The alignment was filtered with a gap filter threshold of 0.8 and an entropy threshold of 0.1. A phylogenetic tree was then constructed at 60% similarity. Alpha and Beta diversity analyses were conducted at a depth of 100 sequences and all samples with sequence counts below 100 were excluded, resulting in 10 controls and 11 patients. Alpha metrics included phylogenetic diversity, the number of observed species and Shannon and Chao 1 indices. Beta diversity was evaluated using both weighted and unweighted UniFrac distances. Statistical significance of diversity comparisons was determined in QIIME using a Wilcoxon rank-sum test.

## Results

### Sample population

DNA was extracted from the stool samples of 49 ME/CFS patients and 39 healthy individuals. After amplification of the 18S rRNA gene, sequencing and processing, we identified eukaryotic sequences in 17 patients with ME/CFS and 17 healthy individuals for further analysis. Within the healthy population, there were 16 females and one male, while within the ME/CFS population there were 13 females and four males (Table 1). The average age was similar between both groups at 44.6 ± 10.9 in controls and 52 ± 11.9 in patients (Table 1). Average Body Mass Index (BMI) was nearly identical at 27.4 ± 4.5 in controls and 26.8 ± 4.7 in patients (Table 1). 11 of 17 ME/CFS patients reported gastrointestinal symptoms, compared to six of 17 healthy individuals (Table 1). We additionally had Bell Scale ratings for 15 patients, with scores ranging from 10 to 50 out of 100 in all individuals (Table 1).

**Table 1:** Subject characteristics. Characteristics of study population with adequate sequence data and subgroup used for diversity calculations. ME/CFS patient and healthy control subjects broken down by sex, comparison of average age and BMI, and number of individuals reporting gastrointestinal symptoms. ME/CFS patients are additionally broken down by Bell Disability Scale scores.

		Taxa abundance comparisons		Diversity subgroup	
		Controls (*n* = 17)	ME/CFS (*n* = 17)	Controls (*n* = 10)	ME/CFS (*n* = 11)
Males		1	4	1	4
Females		16	13	9	7
Age		44.6 ± 10.9	52 ± 11.9	43.6 ±13.6	54 ± 11.6
BMI		27.4 ± 4.5	26.8 ± 4.7	27.7 ± 5.2	26.2 ± 4.9
Gastrointestinal symptoms		6	11	3	8
Bell Scale	10		1		0
	20		4		3
	30		2		1
	40		5		3
	50		3		2
	N/A	17	2	10	2

### Sequencing results and OTU picking

Sequencing yielded 12,430,061 raw reads. After quality control and library splitting, a total of 3,401,011 reads remained. Open-reference OTU picking resulted in 2015 unique OTUs with a total of 3,384,926 OTUs. After removing bacteria, archaea, plant and mammal OTUs from further analysis, as well as any OTUs found in negative controls, 484 unique OTUs and 68,214 total OTUs remained. Additional OTUs that were not found in the constructed phylogenetic tree or were determined to have errors in classification were removed, resulting in 64,760 total OTUs and 310 unique OTUs. Finally, any OTUs not represented by a minimum of 5 sequences in an individual were removed for the purpose of comparing relative taxa abundance. This left a total of 34 individuals (17 patients and 17 controls) for taxa analysis, as well as 310 unique OTUs and 63,847 total OTUs. The average OTUs per individual was 1,878 and the standard deviation was 6,435.

### Identified taxa

**Figure 1:**
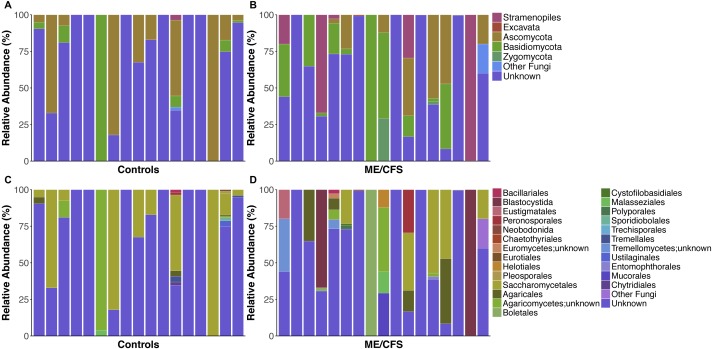
Individual relative taxa abundances in healthy controls and ME/CFS patients. (A) Relative abundance of eukaryotes observed at the phyla level for 17 healthy controls and (B) 17 ME/CFS patients. Each bar represents one individual. Abundances are expressed as percent of total observed OTUs in an individual. Taxa are considered present if represented by a minimum of 5 OTUs. Unknown refers to OTUs with no match in SILVA 119 database. (C) Relative abundance of eukaryotes observed at the level of order for 17 healthy controls and (D) 17 ME/CFS patients. In cases where OTUs were not classified at the level of order, names indicate lowest identified taxa level followed by unknown.

**Figure 2:**
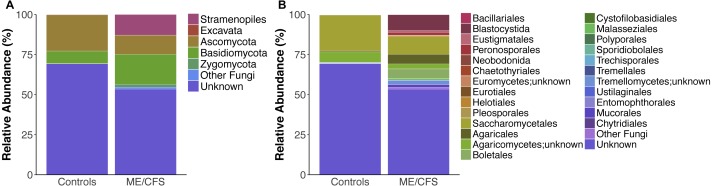
Average relative taxa abundances in healthy controls and ME/CFS patients. (A) Average relative abundance of taxa at the level of phyla in healthy controls versus ME/CFS patients. (B) Average relative abundance of taxa at the level of order in healthy controls versus ME/CFS patients.

On average, fungi were the most abundant taxa identified in our population (32.1%). Within fungi, the phylum *Ascomycota* was the most abundant (17.3%), followed by *Basidiomycota* (13.3%) and *Zygomycota* (0.9%). An average of 0.7% were fungi, but belonged either to another phylum or were unclassified at the phyla level. For simplicity, these were grouped as “Other Fungi” (Figs. 1 and 2). A small proportion of OTUs were classified as *Stramenopiles* (6.6%) and one ME/CFS patient contained 0.8% *Excavata* (Figs. 1 and 2). An average of 61.4% of OTUs per individual were unclassified at any level after BLAST against the SILVA 119 database and termed “Unknown” (Figs. 1 and 2). Additional OTUs could not be classified to consensus at lower levels of taxonomy. Thus, in many cases, analysis was restricted to higher levels of phylogeny.

Nearly all of the fungal OTUs identified as *Ascomycota* were classified in the *Saccharomycetales* order (97%). Additional observed *Ascomycota* were members of class *Euromycetes* and the orders *Pleosporales, Chaetothyriales, Eurotiales* and *Helotiales*. Within *Basidiomycota*, the majority of OTUs belonged to the *Agaricomycetes* class (83.7%), while much of the remainder belonged to *Tremellomycetes* (9.4%), followed by, *Microbotryomycetes*, *Exobasidiomycetes*, and *Ustilaginomycetes*. Most OTUs within *Agaricomycetes* were members of the *Agaricales* order, but others belonged to the orders *Boletales*, *Polyporales*, and *Trechisporales*. Similarly, most OTUs from *Tremellomycetes* were from order *Tremellales*, but one individual also had OTUs from *Cystofilobasidiales* (Fig. 1D). *Zygomycota* consisted mainly of order *Mucorales* (93.9%), with a small proportion of *Entomophthorales* (6.1%).

Among *Stramenopiles*, almost all OTUs were of the *Blastocystida* order (74.9%). Less abundant *Stramenopiles* belonged to *Peronosporales* (14.1%), *Pleurosigma* (2.1%), and *Eustigmatales* (9%).

Analysis of differences in relative abundance at lower levels of taxonomy was limited due to many unclassified reads. In particular, many of the reads assigned to the *Saccharomycetales* order were not classified further.

### Specific taxa abundances of gut eukaryotes in ME/CFS patients are not significantly altered from healthy controls

We utilized the entire dataset for comparison of taxa abundance. However, an OTU was only considered present in an individual sample if it was represented by a minimum of five OTUs, as previously recommended (Parfrey, Walters & Knight, 2011). The composition of eukaryotic microorganisms was relatively unique from one individual to another. Despite this, among identified OTUs, most individuals had a majority of fungal species. Differences in abundances of specific eukaryotes between ME/CFS individuals and healthy individuals did not reach statistical significance at any level of taxonomy.

**Figure 3:**
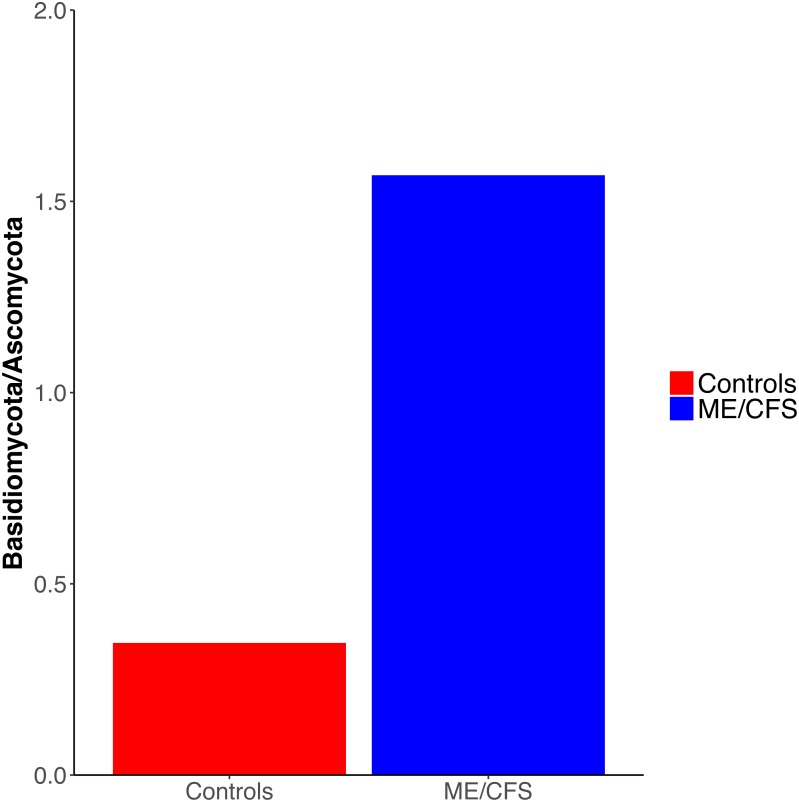
Ratio of Basidiomycota to Ascomycota fungal phyla. Ratio of the average abundance of Basidiomycota to Ascomycota fungal phyla in 17 healthy controls versus 17 ME/CFS patients.

Overall, healthy controls had a slightly lower average relative abundance of fungi compared to ME/CFS patients (30.6% to 33.6%) (Fig. 2A). At the phylum level, the abundance of *Ascomycota* was decreased from healthy controls to ME/CFS patients (22.6% versus 11.9%) (Fig. 2A). This was reflected in a corresponding increase in the fungal phylum *Basidiomycota* from controls to patients (7.8% to 18.7%) (Fig. 2A). As a result, there was a nonsignificant increase in the ratio of *Basidiomycota* to *Ascomycota* from healthy controls to patients (0.3 vs 1.6) (Fig. 3). The fungal phylum *Zygomycota* was also increased in ME/CFS (0% versus 1.8%), but this was largely driven by merely 2 patients (Figs. 1B and 2A). Similarly, an increase in *Stramenopiles* from 0.2% in controls to 12.9% in ME/CFS was mainly due to a high abundance of *Blastocystis* in two patients (Figs. 1D and 2A). One ME/CFS patient did contain a small proportion of *Excavata* (0.8%), classified as order *Neobodonida* (Figs. 1B and 1D).

Within *Ascomycota*, the predominant difference was at the level of order, with the relative abundance of *Saccharomycetales* decreased from healthy individuals to ME/CFS patients (22.5% to 11%) (Fig. 2B). Within *Basidiomycota*, a number of increases in relative abundance contributed to the overall higher abundance. Abundance of the class *Agaricomycetes* increased from healthy individuals to ME/CFS patients (6.9% to 15.3%). This included increased abundances of the orders *Agaricales* (0.5% to 6%), *Boletales* (0% to 6%) and *Polyporales* (0% to 0.14%) (Fig. 2B). *Basidiomycota* class *Tremellomycetes* also increased from healthy controls to ME/CFS patients overall (0.3% to 2.6%). Specifically, this was due to *Tremellomycetes;unknown* (0.0% versus 2.5%) and *Cystofilobasidiales* (0% to 0.08%) (Fig. 2B). Interestingly, a small proportion of OTUs were indentified as *Tremellales* in healthy individuals (0.3%), but none were identified in ME/CFS patients (Fig. 2B). ME/CFS patients also had increased abundance of *Malasseziales* (0.2% to 0.9%), but this order was only identified in one healthy individual and one ME/CFS patient (Figs. 1C–1D and 2B). The *Basidiomycota* orders of *Sporidiobolales* and *Ustilaginales* were only present in healthy controls and at a very low level (0.1% and 0.2% respectively) (Fig. 2B). In each case, these low abundances were driven by a single individual (Fig. 1C). The increase in *Zygomycota* abundance in ME/CFS patients was due to slight increases in the relative abundance of *Entomophthorales* (0% to 0.1%) and *Mucorales* (0% to 1.7%) due to single patients containing OTUs in these orders (Figs. 1D and 2).

In addition to an increase in the abundance of *Blastocystis*, ME/CFS patients had a higher average relative abundance of the *Stramenopile* orders *Pleurosigma* (0.1% to 0.2%), *Eustigmatales* (0% to 1.2%) and *Peronosporales* (0.1% to 1.7%) (Fig. 2B).

Finally, ME/CFS patients exhibited a lower average relative abundance of OTUs that were unknown after BLAST against the SILVA 119 database (69.2% to 53.4%) (Fig. 2A). Among specific OTUs that were unidentified, there were no significant differences in relative abundance between ME/CFS patients and healthy individuals.

### Gut eukaryotic diversity in ME/CFS does not differ from healthy controls

To evaluate diversity levels in ME/CFS patients versus healthy individuals, we utilized metrics of alpha and beta diversity. Alpha rarefaction was performed at a depth of 100 sequences, which allowed us to include 11 ME/CFS patients and 10 healthy controls for analysis. The characteristics of these subpopulations were similar to those of the entire population (Table 1). The 10 healthy individuals had an average age of 43.6 ± 13.6 and average BMI of 27.7 ± 5.2, while the 11 ME/CFS patients had an average age of 54 ± 11.6 and average BMI of 26.2 ± 4.9 (Table 1). There were nine females and one male in the healthy control group and seven females and four males in the patient group (Table 1). Only three healthy individuals reported gastrointestinal symptoms, compared to 8 ME/CFS patients (Table 1).

**Figure 4:**
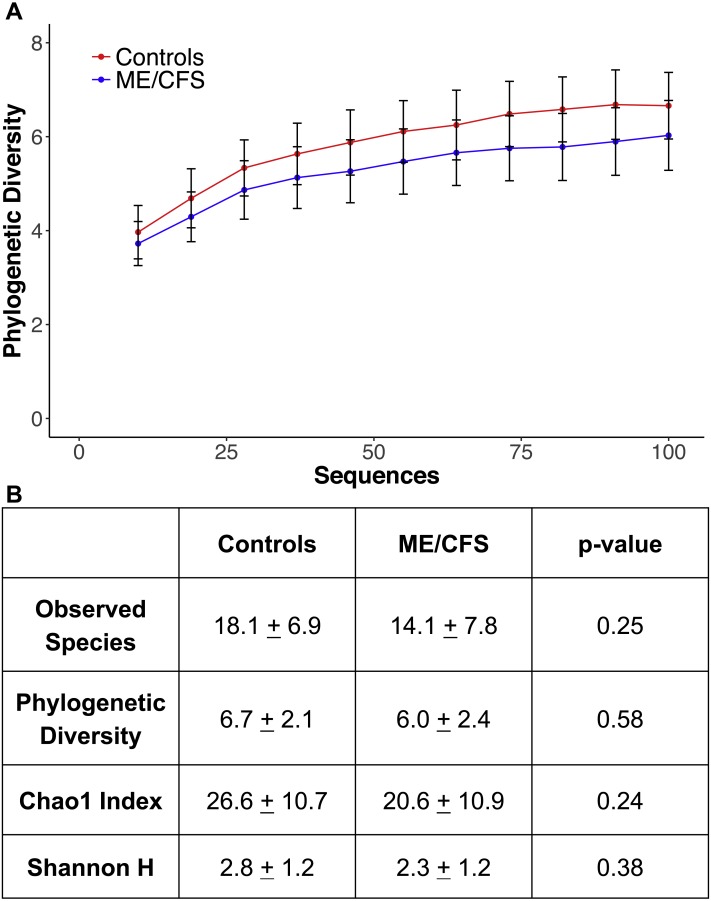
Alpha rarefaction and diversity indices. (A) Phylogenetic diversity as calculated during alpha rarefaction up to maximum depth of 100 sequences in 11 ME/CFS patients and 10 healthy controls. Error bars represent standard error of the mean. (B) Average observed species, Shannon index, Chao 1 index and phylogenetic diversity of healthy controls and ME/CFS patients plus or minus standard error of the mean. Statistical significance was evaluated with a Wilcoxon-rank sum test.

At a depth of 100 sequences, rarefaction revealed that phylogenetic diversity approached an asymptote in both ME/CFS patients and healthy individuals (Fig. 4A). Although there was a small decrease in phylogenetic diversity from controls to ME/CFS patients (6.7 ± 2.1 vs 6.0 ± 2.4), it was not statistically significant (*p* = 0.58) (Fig. 4). We also calculated several other metrics of alpha diversity, including Chao 1, Shannon and number of observed species. Again, these values did not reveal a statistically significant difference in the alpha diversity, despite consistently lower mean values in ME/CFS patients versus healthy controls (Fig. 4B).

**Figure 5:**
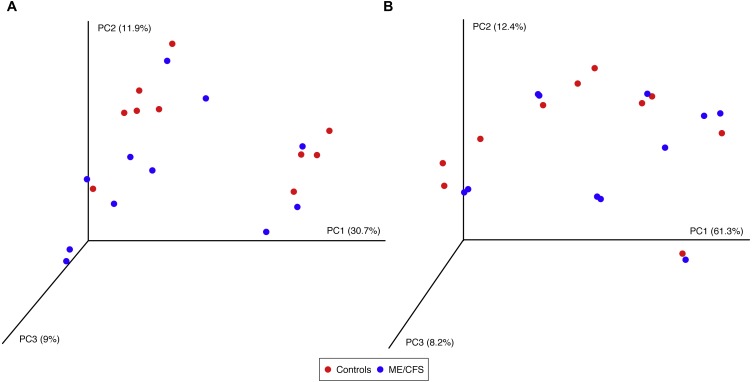
Principal Coordinates Analysis. (A) Principal Coordinates Analysis of unweighted UniFrac distances for 11 ME/CFS patients and 10 healthy controls at an even depth of 100 sequences. (B) Principle Coordinates Analysis of weighted UniFrac distances for 11 ME/CFS patients and 10 healthy controls at an even depth of 100 sequences.

Using the same depth of rarefaction and individuals, we also evaluated beta diversity in our population. We computed both unweighted and weighted UniFrac distance matrices for the evenly sampled population and visualized the results in Principle Coordinates Analysis plots (Fig. 5). Neither plot revealed clustering of individuals based on disease, nor based on the additional variables for which we had collected information (data not shown).

## Discussion

Compared to studies of prokaryotes in the human gut microbiome, studies of eukaryotes in the human gut are limited. Many existing reports of gut eukaryotes use methods other than high throughput sequencing. We sought to achieve characterization of eukaryotes in the gut of ME/CFS patients previously tested for bacterial composition and to compare eukaryotic diversity and composition to healthy individuals. Many of the taxa observed in our population have been identified in the human gut in previous studies and in some cases, associated with disease.

Fungi belonging to the *Ascomycota*, *Basidiomycota* and *Zygomycota* phyla have been previously observed in the human gut (Hamad, Raoult & Bittar, 2016). *Saccharomycetales* are commonly identified *Ascomycota* in the human gut and were observed in our population (Figs. 1C–1D and 2B) (Hamad, Raoult & Bittar, 2016; Li et al., 2014; Parfrey, Walters & Knight, 2011; Sokol et al., 2017). *Malessiziales*, members of *Basidiomycota*, were present in one healthy control and one ME/CFS patient and have been previously found in fecal samples and associated with IBD (Figs. 1C–1D) (Hallen-Adams & Suhr, 2017; Sokol et al., 2017). *Mucorales*, members of *Zygomycota*, are commonly identified and can cause infections in humans, particularly when the immune system is compromised (Hamad, Raoult & Bittar, 2016; Mendoza et al., 2014). We identified *Mucorales* in one ME/CFS patient, but were unable to classify this OTU further (Fig. 1D). Another *Zygomycota* order, *Entomophthorales*, was present at low abundance in one ME/CFS patient, who did report gastrointestinal symptoms (Fig. 1D). Species of *Entomophthorales* are also capable of causing infections in humans (Mendoza et al., 2014).

*Blastocystis* are thought to be the main, if not only, *Stramenopile* present in the human gut (Hamad, Raoult & Bittar, 2016). We observed *Blastocystis* in three ME/CFS patients, comprising the majority of OTUs in two of those patients (Fig. 1D). This relatively low incidence of *Blastocystis* is surprising in comparison to other studies of human gut eukaryotes (Parfrey, Walters & Knight, 2011; Scanlan & Marchesi, 2008). Interestingly, all three of the ME/CFS patients with detectable *Blastocystis* reported gastrointestinal symptoms.

Challenges associated with characterization of eukaryotes in the human gut microbiome include their low abundance and diversity, as well as the influence of host and diet DNA. DNA belonging to eukaryotic microorganisms is only a small component of that isolated from fecal samples, limiting the number of high quality eukaryotic reads. Although the SILVA database makes it possible to identify eukaryotes based on the 18S rRNA hypervariable regions, the lack of identification of many reads in our study suggests that 18S databases are still incomplete. Lower level classification can be impossible, particularly in fungi, using the 18S rRNA gene. For this reason, sequencing the internal transcribed spacers, found in between eukaryotic rRNA genes, can be advantageous to improve species level resolution of fungi. Eukaryotic identification is also challenging due to changing and complex taxonomic classification systems (Dollive et al., 2012; Hamad, Raoult & Bittar, 2016).

Previous studies of human gut eukaryotes have encountered similar difficulties. Parfrey, Walters & Knight (2011) reported low sequence abundance, but also showed that a sequence depth of 150 sequences of the 18S rRNA gene was sufficient to achieve maximum diversity in human fecal samples. Furthermore, they showed a wide variety of unique eukaryotic communities from one individual to another. This has likely contributed to the differences in reports of human gut eukaryotes and makes comparisons of taxa abundance between groups of subjects challenging.

The phylum *Basidiomycota* is influenced by diet, as it contains edible mushrooms within order *Agaricales*, which were commonly identified in our subjects (Figs. 1C–1D and 2B) (Hoffmann et al., 2013). Similarly, abundances of *Saccharomycetales* may be influenced by diet (Hallen-Adams & Suhr, 2017; Hoffmann et al., 2013). While our data show a high level of *Saccharomycetales*, as previously shown, we did not see a high abundance of *Candida* species within this order (data not shown). This may be due to an inability to classify many of the *Saccharomycetales* beyond the level of order, as well as the difficulty of distinguishing *Saccharomyces* and *Candida* from one another (Dollive et al., 2012). We saw little *Blastocystis* across our data set, which is a common commensal organism in the human gut, and we did not identify the *Dientamoeba* or Nematodes that others have found (Parfrey, Walters & Knight, 2011; Scanlan & Marchesi, 2008). These limits may be due to the primers used or DNA extraction method, which may not have been able to lyse particular cells or spores (Dollive et al., 2012; Parfrey, Walters & Knight, 2011).

Interestingly, we did observe a shift in the relative abundance of *Ascomycota* and *Basidiomycota* from healthy individuals to ME/CFS patients, although these changes were not statistically significant (Fig. 3). *Ascomycota* and *Basidiomycota* have previously been found to be inversely correlated with one another (Hoffmann et al., 2013; Sokol et al., 2017). This is consistent with our results, in that ME/CFS patients exhibit both decreased *Ascomycota* and increased *Basidiomycota* (Fig. 2A). Furthermore, Sokol et al. (2017) demonstrated that the ratio of *Basidiomycota* to *Ascomycota* was statistically significantly increased in patients with IBD during flares, but returned near levels seen in healthy controls when patients were in remission, suggesting a link between this ratio and inflammation. An additional study of patients with Crohn’s disease also revealed increased *Basidiomycota* abundance and decreased *Ascomycota* compared to healthy individuals (Li et al., 2014). Thus, the increase in this ratio in ME/CFS versus healthy individuals is consistent with an inflammatory state in patients. However, we cannot rule out the influence of dietary differences on these ratios.

As we have previously shown a decrease in prokaryotic diversity and bacterial dysbiosis in ME/CFS patients, we may have expected significant alterations in diversity and eukaryotic abundance in patients (Giloteaux et al., 2016). Although not significant, we did demonstrate a small decrease in eukaryotic diversity in ME/CFS patients compared to healthy controls (Fig. 4). Eukaryotes in the gut microbiome have been demonstrated to correlate with prokaryotes (Audebert et al., 2016; Chehoud et al., 2015; Hoffmann et al., 2013; Sokol et al., 2017). Correlations between bacterial and eukaryotic diversity in the gut are inconsistent, as are reports of changes in eukaryotic diversity during inflammatory disease. Increased diversity of eukaryotes has been associated with Crohn’s disease in two separate studies (Li et al., 2014; Ott et al., 2008). Conversely, decreased fungal diversity has been demonstrated in patients with ulcerative colitis compared to healthy individuals or those in remission (Sokol et al., 2017). Similarly, pediatric patients with IBD have also been shown to have decreased fungal diversity (Chehoud et al., 2015).

## Conclusions

Our results demonstrate that the ME/CFS gut eukaryotic composition is consistent with an inflammatory state in ME/CFS patients. Among the eukaryotes we identified, there was not a specific gut eukaryote associated with ME/CFS. However, ME/CFS patients did display an increased ratio of average *Basidiomycota* to *Ascomycota* abundance, which has been previously linked to inflammation in IBD. Additionally, ME/CFS patients had a nonsignificant decrease in gut eukaryotic diversity. Our study demonstrates the challenge of comparing the eukaryotic microbiota between a patient and control group due to difficulties in extraction of nucleic acid and identification of sequences, the influence of outside factors such as diet, as well as the great individual variation between eukaryotic taxonomic units present even within-group. Despite these challenges, recent findings have underscored the role of eukaryotes in the gut ecosystem, as well as in disease. Given the previous evidence suggesting a link between ME/CFS and pathogenic eukaryotes, characterization of ME/CFS patient and healthy control gut eukaryotes is valuable, even though our population size was limited. Our data is consistent with the inflammatory state within the gut that was documented in prior studies of the ME/CFS bacterial microbiome. A key issue for future research is determining what causes inflammation in ME/CFS.

## Additional Information and Declarations

### Data Availability

The following information was supplied regarding data availability: Sequence data is available in the European Nucleotide Archive of the European Bioinformatics Institute under accession number PRJEB23827.
